# Disparities in HIV Screening among Pregnant Women – El Salvador, 2011

**DOI:** 10.1371/journal.pone.0082760

**Published:** 2013-12-09

**Authors:** Charbel El Bcheraoui, Ana I. Nieto Gómez, María A. Dubón Abrego, Marielle C. Gagnier, Madeline Y. Sutton, Ali H. Mokdad

**Affiliations:** 1 Institute for Health Metrics and Evaluation, University of Washington, Seattle, Washington, United States of America; 2 STD-HIV/AIDS Program, Ministry of Health, San Salvador, El Salvador; 3 First Level of Attention, Ministry of Health, San Salvador, El Salvador; 4 Department of Obstetrics and Gynecology, Morehouse School of Medicine, Atlanta, Georgia, United States of America; University of North Carolina School of Medicine, United States of America

## Abstract

**Objectives:**

To provide an accurate estimate of antenatal HIV screening and its determinants among pregnant women in El Salvador and help local authorities make informed decisions for targeted interventions around mother-to-child transmission (MTCT).

**Methods:**

A total sample of 4,730 women aged 15-49 years were interviewed from a random sample of 3,625 households. We collected data on antenatal care services, including HIV screening, during last pregnancy through a pre-established questionnaire. We used a backward elimination multivariate logistic regression model to examine the association between HIV screening and sociodemographic and health care-related factors.

**Results:**

A total of 2,929 women were included in this analysis. About 98% of participants reported receiving antenatal care, but only 83% of these reported being screened for HIV. Screening was lower in geographic areas with higher HIV incidence and ranged from 69.1% among women who were not seen by a physician during antenatal care, to 93.7% among those who attended or completed college. Odds for screening varied also by age, employment status, household economic expenditure, possession of health care coverage, health care settings, and number of antenatal care visits.

**Conclusions:**

We found disparities in HIV screening during antenatal care at the environmental, social, demographic, and structural levels despite a high uptake of antenatal care in El Salvador. Our findings should urge health authorities to tailor and enhance current strategies implemented to eliminate MTCT and reduce inequities and HIV morbidity among women in El Salvador.

## Introduction

HIV was the sixth leading cause of death among women of reproductive age, 15 - 49 years, in El Salvador in 2010 [1], and has persisted among the 10 leading causes of death for all ages among persons in El Salvador between 1999 and 2009 [2–8]. The number of people living with HIV/AIDS in El Salvador has been rising since the late 1990s and currently ranges between 28,000 and 34,000 [9,10]. Underreporting of HIV cases has been repeatedly cited as a major problem [11,12]. With an HIV prevalence of 4.5 - 5.5 cases per thousand residents [9,10], El Salvador has the third highest prevalence in Latin America. About 2,000 children aged 0 – 14 years are known to be currently living with HIV in El Salvador [10]. In addition, mother-to-child transmission (MTCT) accounts for 6% of new cases of the various HIV transmission modes, the second highest MTCT attributable risk percentage among Latin American countries [10]. However, national authorities report a considerably lower fraction, not exceeding 0.2%, of new HIV cases attributable to MTCT, whereas 7% of newborns from HIV-infected mothers are also HIV-infected. Health is a big focus of the Salvadorian government [13], and health authorities have adopted a nationwide program for prevention of MTCT (PMTCT), with a goal of having an HIV-free newborn generation by 2015 [12] or reducing MTCT from the current 7% to only 2% [9]. The program focuses on diagnosing and treating HIV-positive pregnant women and is making great progress to date [9,12]. 

The World Health Organization considers HIV screening to be the first and most crucial step in PTMCT, and the U.S. Centers for Disease Control and Prevention (CDC) recommends widespread HIV screening in health care settings and early universal screening for HIV for pregnant women [14–17]. The current national guidelines around PMTCT in El Salvador, published in 2012, recommend HIV screening for every pregnant woman within the first 3 months of pregnancy. An initial voluntary, confidential, and free-of-charge rapid HIV test is offered during the first antenatal care visit. Positive tests are sent for confirmation by ELISA 3^rd^ or 4^th^ generation. Women are informed of their HIV status four weeks later or earlier, during the next antenatal care visit. Women who are confirmed as HIV-infected are offered universal free-of-charge antiretroviral treatment (ART) as soon as possible and are given condoms and counseled on avoiding unsafe sex. They are also offered substitute milk for their infant for up to 18 months of life. ART is also recommended for up to a month and a half for these infants. Pregnant women are also offered HIV screening at labor if no previous screening was performed. In the case where women are found HIV-infected at or close to labor, specific ART guidelines are also applied to prevent MTCT [18]. 

It has been reported that more than 97% of pregnant women were tested for HIV in El Salvador in 2007 [12,19], an even higher rate than the 96% reported in Ontario, Canada, for 2010 [20]. If this quasi-universal HIV screening for pregnant women is valid, it could be one of the biggest achievements in antenatal care service delivery. Given that this antenatal HIV screening is not mandatory, while recognizing its relevance to women and children’s health, there could be an overestimation of the true percent of screened pregnant women, especially with an absence of details on the data source for the reported estimate [19]. We also expect that variations in the screening uptake exist by sociodemographic and health care characteristics. To help local authorities make informed decisions for targeted interventions around PMTCT, we analyzed data on HIV screening at last pregnancy from a representative sample of childbearing-age women in El Salvador as part of the Salud Mesoamerica (SM2015) Initiative. Specifically, our objectives were 1) to estimate the percent of pregnant women screened for HIV through antenatal care, and 2) to identify screening predictors and barriers at the sociodemographic and health care level.

## Methods

### Ethics statement

The consenting procedures for this study were approved by the University of Washington’s Institutional Review Board and the Ministry of Health in El Salvador. Participants were asked to sign a written consent form prior to taking part in the study. 

SM2015 is an innovative public/private partnership that seeks to reduce health equity gaps faced by those living in extreme poverty in Mesoamerica. The principal objective of the SM2015-El Salvador Baseline Household Survey was to collect baseline data on household characteristics, household expenditures, and numerous reproductive health, maternal and neonatal health, immunization, and nutrition indicators (including physical measurements) related to the strategic areas of the initiative in El Salvador. 

The sample for the SM2015-El Salvador Baseline Household Survey was designed to provide estimates of the coverage of key health interventions and indicators among the lowest wealth quintile of the population. The primary administrative units in El Salvador are departments and municipalities. El Salvador comprises 14 departments and 262 municipalities. We identified 14 municipalities from eight departments in which to conduct the SM2015-El Salvador Baseline Household Survey for the initiative on the basis of their high concentration of residents in the country’s lowest wealth quintile. The 14 targeted municipalities include 523 segments. From these, 136 segments were randomly selected using systematic sampling with probability proportional to size based on the number of occupied households in the 2007 El Salvador Census. Within each selected segment, we conducted our own census in order to identify eligible women and children for the survey in the selected segments. A total of 14,230 households were captured in our census. Of these, a random sample of 3,935 households were visited, of which a total of 3,625 were interviewed (intended sample = 3,800). We created three sets of sampling weights at the levels of selected households, women, and children. The weights incorporated the probability of the segment being selected (according to the 2007 census data), the probability of the household being selected (using our own census), and the proportion of women and children surveyed in the selected households. In this analysis, we included women who satisfied all of the three following conditions: 1) ever gave birth, 2) had a pregnancy that resulted in a live birth over the last five years, and 3) received at least one antenatal care visit during that pregnancy. 

Consenting women were interviewed face-to-face by trained field surveyors. Data were collected on paper questionnaires and entered on computers by trained data entry personnel. Women were asked questions about different health-related topics including antenatal, delivery, and postpartum care. For antenatal care, women were asked about types of services included in their visits, including if they had received an HIV test as part of their antenatal care. Since data were self-reported, we analyzed data from the most recent pregnancy to account for recall bias. We also checked whether rates of HIV screening differed when we restricted the sample to pregnancies in the last two years only. 

We first calculated rates of HIV screening. Second, we used chi-squared tests to measure association between women’s sociodemographic characteristics, such as geographic area of residency, age group, educational level, marital and employment status, household economic level measured by total monthly expenditure, and possession of medical insurance coverage, and their health care-related characteristics, such as the number of antenatal care visits, type of health care provider and setting, and rates of HIV screening. The geographic areas where women lived were redistributed according to their HIV incidence in 2010 to analyze uptake of HIV screening in parallel with HIV incidence risk. Hence, the eight departments were redistributed into three areas. The first area, La Paz, had an HIV incidence greater than 3 per 10,000 population; the second area, which included Ahuachapan, Cuscatlan, and La Libertad, had an HIV incidence of 2 - 2.9 per 10,000 population; the third area, which included Cabanas, La Union, Morazan, and San Vincente, had an HIV incidence of less than 2 per 10,000 population [21]. Third, characteristics that proved associated with rates of HIV screening at *p* ≤0.05 were entered in a backward elimination multivariate logistic regression model. Adjusted odds ratios (AORs) for having received an HIV test and their 95% confidence intervals (CI) were calculated. Significance of association was considered for 95% CI, excluding the value of one. We used SAS 9.2 (SAS Institute Inc., Cary, NC, USA) to analyze the weighted data and account for the complex sampling design.

## Results

**Figure 1:**
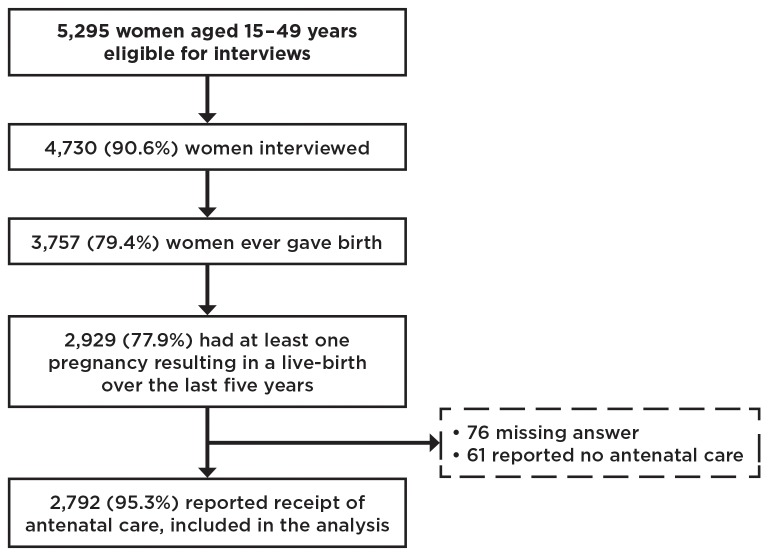
Distribution of women from those eligible to be interviewed to those included in the analysis sample.

Between March and June 2011, we interviewed a total of 4,730 women aged 15 - 49 years. Of these, 3,757 women (79.4%) had ever given birth and 2,929 (77.9%) of them had had at least one pregnancy over the last five years. Sociodemographic characteristics of these women are detailed in Table 1. Use of antenatal care was high, as 2,792 women (95.3%) reported receipt of at least one antenatal care visit during their last pregnancy; of the 137 remaining women, 76 did not answer the question about receipt of antenatal care, and 61 reported not having received antenatal care during pregnancy. Data related to HIV screening were analyzed for the 2,792 women who reported receipt of at least one antenatal care visit during their last pregnancy. Figure 1 details the sample of women included in our analysis. 

**Table 1:** Frequencies and percentages of sociodemographic characteristics and antenatal care visits at last pregnancy of parous participants (sample size = 2,929).

**Factor**	**Categories**	**Frequencies**	**% unweighted *^a^***	**% weighted *^a^***
**Geographic area by HIV incidence**	< 2 / 10,000 population	2065	70.5	71.2
	2 - 2.9 / 10,000 population	723	24.7	24.2
	≥ 3 / 10,000 population	141	4.8	4.6
	Total	2929	100.0	100.0
**Age groups**	15 - 19	327	11.2	11.2
	20 - 29	1472	50.2	49.4
	30 - 39	905	30.9	31.0
	40 - 49	225	7.7	8.4
	Total	2929	100.0	100.0
**Educational level**	Literacy course or no education	330	11.3	11.4
	Primary	1552	53.1	53.1
	Secondary	877	30.0	29.7
	University	165	5.6	5.8
	Total	2924**^*b*^**	100.0	100.0
**Marital status**	Currently in a relationship	2163	74.9	74.4
	Currently not in a relationship	726	25.1	26.6
	Total	2924**^*b*^**	100.0	100.0
**Employment status**	Currently employed	332	11.3	11.4
	Currently unemployed	2592	88.7	88.6
	Total	2889**^*b*^**	100.0	100.0
**Household economic level**	Lowest monthly expenditure tertile	981	34.2	33.8
	Average monthly expenditure tertile	945	32.9	32.9
	Highest monthly expenditure tertile	945	32.9	33.3
	Total	2871**^*b*^**	100.0	100.0
**Received at least one antenatal care visit**	Yes	2792	97.9	97.9
	No	61	2.1	2.1
	Total	2853**^*b*^**	100.0	100.0

^a^ Unweighted percentages are based on the actual frequencies from the analyzed sample. Weighted percentages are obtained after weighting the data using the post-stratification weights detailed in the methods section.

^b^ Totals that do not add up to 2,929 are due to missing values.

Among the 2,792 women included in the analysis, 83% reported being screened for HIV during their last pregnancy as part of antenatal care — only two participants had a missing answer when asked about receiving HIV screening. This 83% rate increased by less than 2% when analysis was restricted to pregnancies over the last two years; among the 1,324 women who had their last pregnancy within two years and received antenatal care, 1,117 (84.7% weighted percent) were screened for HIV.

**Figure 2:**
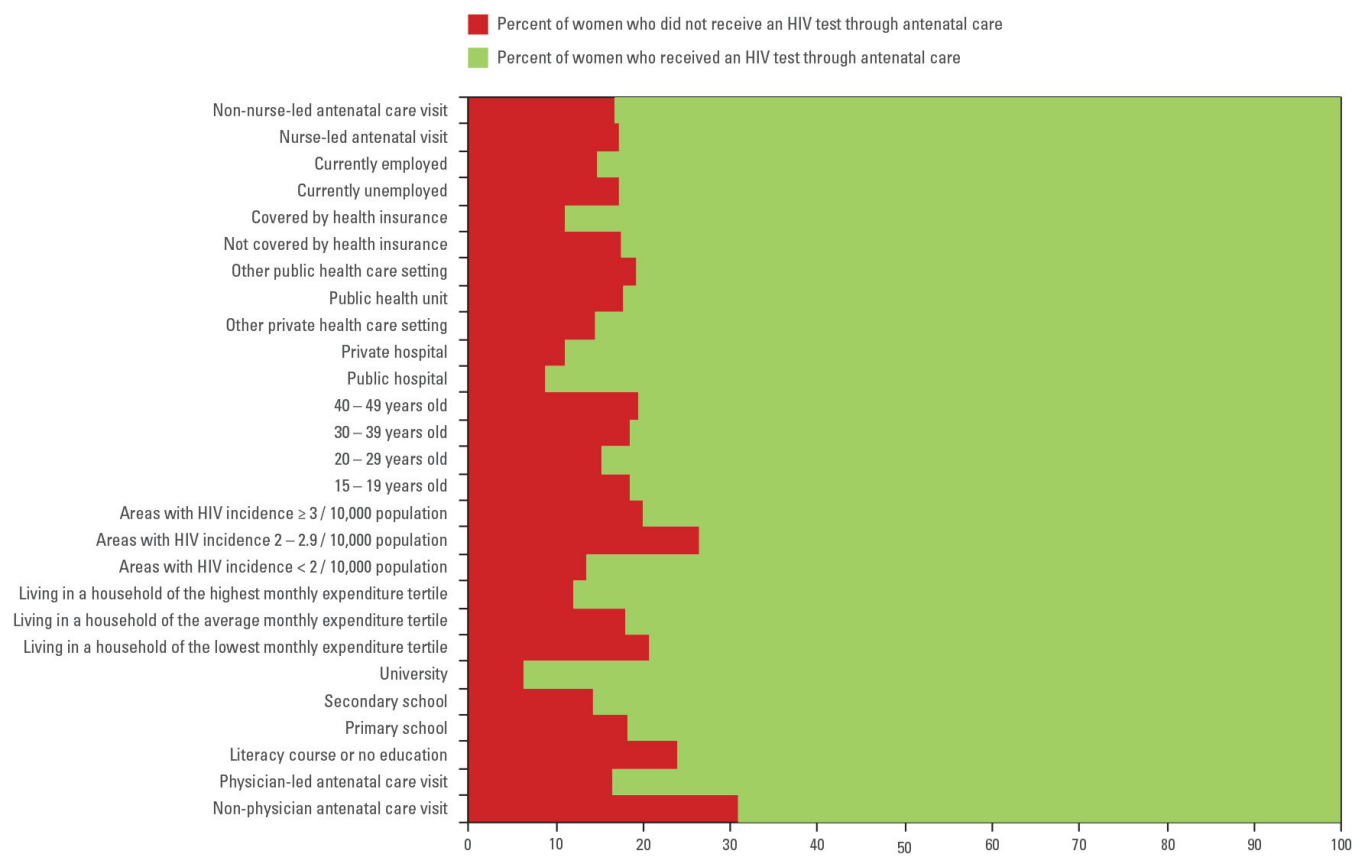
Distribution of HIV screening during pregnancy by socio-demographic and health care-related characteristics.

HIV screening uptake varied from a low of 69.1% among women who were not seen by a medical doctor during antenatal care, to a high of 93.7% among women who attended college (Figure 2). Variation of HIV screening was statistically significant through all sociodemographic and health care-related characteristics, except when the health care provider type was a nurse aide. Being seen by a nurse aide during antenatal care was not associated with receiving an HIV test (Table 2).

**Table 2:** Weighted chi-squared test of receiving an HIV test as part of antenatal care at last pregnancy by socio-demographic and health care-related characteristics.

		**Received and HIV test as part of antenatal care**			
**Factor**	**Categories**	**No (%)**	**Yes (%)**	**Chi^2^**	**P value**
**Geographic area by HIV incidence**	< 2 / 10,000 population	4717 (13.6)	29983 (86.4)	1060.3	< 0.0001*
	2 - 2.9 / 10,000 population	3193 (26.4)	8881 (73.6)		
	≥ 3 / 10,000 population	440 (20.0)	1760 (80.0)		
	Total	8350 (17.0)	40624 (83.0)		
**Age groups**	15 - 19	1038 (18.5)	4576 (81.5)	95.0	< 0.001*
	20 - 29	3772 (15.4)	20710 (84.6)		
	30 - 39	2830 (18.5)	12459 (81.5)		
	40 - 49	778 (19.5)	3213 (80.5)		
	Total	8418 (17.0)	40956 (83.0)		
**Educational level**	Literacy course or no education	1269 (24.1)	3989 (75.9)	528.3	< 0.001*
	Primary	4816 (18.3)	21551 (81.7)		
	Secondary	2118 (14.3)	12662 (85.7)		
	University	182 (6.3)	2708 (93.7)		
	Total	8385 (17.0)	40910 (83.0)		
**Marital status**	Currently in a relationship	6277 (17.4)	29691 (82.5)	11.9	< 0.001*
	Currently not in a relationship	2045 (16.1)	10651 (83.9)		
	Total	8322 (17.3)	40342 (82.7)		
**Employment status**	Currently employed	825 (14.7)	4804 (85.3)	25.6	< 0.001*
	Currently unemployed	7577 (17.3)	36090 (82.7)		
	Total	8402 (17.0)	40894 (83.0)		
**Household economic level**	Lowest monthly expenditure tertile	3428 (20.8)	13053 (79.2)	459.0	< 0.001*
	Average monthly expenditure tertile	2867 (18.0)	13052 (82.0)		
	Highest monthly expenditure tertile	1928 (12.0)	14080 (88.0)		
	Total	8224 (17.0)	40185 (83.0)		
**Has medical insurance**	Yes	485 (11.2)	3835 (88.9)	113.9	< 0.001*
	No	7914 (17.6)	36987 (82.4)		
	Total	8399 (17.2)	40822 (82.8)		
**Physician-led antenatal care visit**	Yes	7921 (16.6)	39852 (83.4)	217.1	< 0.001*
	No	479 (30.9)	1074 (69.1)		
	Total	8400 (17.0)	40927 (83.0)		
**Nurse-led antenatal care visit**	Yes	5078 (17.5)	23987 (82.5)	11.0	< 0.001*
	No	3306 (16.3)	16940 (83.7)		
	Total	8385 (17.0)	40927 (83.0)		
**Nurse aide**-**led antenatal care visit**	Yes	3010 (17.2)	14532 (82.8)	0.4	0.5
	No	5374 (16.9)	26379 (83.1)		
	Total	8385 (17.0)	40911 (83.0)		
**Place of antenatal care visit**	Public hospital	454 (8.8)	4730 (91.2)	308.1	< 0.001*
	Public health unit	6322 (17.9)	28910 (82.1)		
	Other public health care setting	1181 (19.2)	4970 (80.8)		
	Private hospital	29 (11.2)	234 (88.8)		
	Other private health care setting	282 (14.5)	1664 (85.5)		
	Total	8269 (17.1)	40509 (82.9)		
**Number of antenatal care visit**	1 - 3 times	721 (21.8)	2582 (78.2)	153.6	< 0.001*
	4 - 6	3012 (19.1)	12766 (80.9)		
	> 6 times	4652 (15.5)	25443 (84.5)		
	Total	8384 (17.0)	40791 (83.0)		

^*^ *p* < 0.05

When all characteristics were adjusted in the multivariate logistic regression, marital status was not sustained in the model, but all other characteristics were maintained (Table 3). 

**Table 3:** Backward elimination multivariate logistic regression for association of HIV screening among pregnant women and sociodemographic and health care-related factors (sample size = 2,633).

**Factor**	**Categories**	**Adjusted odds-ratios**	**95% confidence limits**
**Departments**	HIV incidence < 2 / 10,000 population	Reference	
	HIV incidence 2 - 2.9 / 10,000 population	0.73	0.65 - 0.81*
	HIV incidence ≥ 3 / 10,000 population	0.50	0.48 - 0.53*
**Age groups**	15 - 19	0.87	0.80 - 0.94*
	20 - 29	Reference	
	30 - 39	0.90	0.85 - 0.95*
	40 - 49	0.77	0.70 - 0.84*
**Educational level**	Literacy course or no education	Reference	
	Primary	1.22	1.12 - 1.32*
	Secondary	1.44	1.32 - 1.57*
	University	2.74	2.28 - 3.30*
**Employment status**	Currently unemployed	Reference	
	Currently employed	0.84	0.77 - 0.91*
**Household economic level**	Lowest monthly expenditure tertile	Reference	
	Average monthly expenditure tertile	1.03	0.97 - 1.09
	Highest monthly expenditure tertile	1.39	1.30 - 1.49*
**Medical insurance**	Covered by a health insurance	Reference	
	Not covered by a health insurance	0.64	0.60 - 0.72*
**Number of antenatal care visits**	1 - 3 times	Reference	
	4 - 6 times	1.16	1.05 - 1.27*
	> 6 times	1.52	1.39 - 1.67*
**Physician-led antenatal care visit**	Yes	Reference	
	No	0.60	0.53 - 0.67*
**Nurse-led antenatal care visit**	Yes	Reference	
	No	1.15	1.09 - 1.21*
**Place of antenatal care visit**	Public hospital	Reference	
	Public health unit	0.55	0.49 - 0.60*
	Other public health care setting	0.43	0.38 - 0.49*
	Private hospital	0.50	0.33 - 0.75*
	Other private health care setting	0.35	0.30 - 0.42*

^*^Statistical difference with value of 1 outside the 95% confidence limits

Women were divided into four age-group categories. Compared to women 20 - 29 years old, women of all other age groups had a lower chance to be screened. Chances for screening increased by women’s educational level and for women living in a household of the highest monthly expenditure tertile, compared to women with no education or those who only took a literacy course, and women living in a household of the lowest monthly expenditure tertile. Women who did not benefit from medical insurance coverage had lower chances for being screened compared to those who did.

At the health care level, women had less chance to be screened for HIV if they were not seen by a physician during antenatal care compared to those who were, or if they received antenatal care in health care settings different from a public hospital. Chances of HIV screening increased for women who received a higher number of antenatal care visits and those whose antenatal care visit was not led by a nurse compared to their counterparts.

## Discussion

We found disparities in reported uptake of HIV screening through antenatal care among pregnant women in El Salvador despite high rates of screening in general and an increase in prenatal care use since 2005 [22]. Among our representative sample of Salvadorian women of the lowest wealth quintile, less than 83% reported being screened for HIV during their last pregnancy. This rate is lower than the previously reported 97% for the year 2007 [12,19] and rates reported in other more or less developed countries, such as Canada and Uganda [20,23]. However, it is higher than the 76% of pregnant women served by the Indian Health Services who were screened for HIV in the United States in 2009 [24]. Moreover, this rate is not equal across different categories of women and different health care delivery settings. 

Disparities were significant at the environmental, demographic, social, and structural levels. Rates of HIV screening varied dramatically by geographic areas. While areas with higher HIV incidence should be prioritized for HIV prevention in general, and specifically for PMTCT, women residing in these areas were less likely to be tested. This finding is noteworthy as it points out not only the inequity in health care provision across regions of the same country, but an unmet need in the most affected areas [21]. Advocates of targeted HIV screening usually recommend restricting screening to those who are the most at risk [25,26]. In countries like the United States, where MTCT contributes to less than 0.1% of new cases, the American College of Obstetricians and Gynecologists, the CDC, and the U.S. Preventive Services Task Force recommend a universal, opt-out HIV screening process for pregnant women that is not linked to a risk assessment [15,27,28]. In addition, WHO has recommended universal prenatal screening as part of its multi-pronged, global approach to achieve an AIDS-free generation [14]. Hence, targeting pregnant women for routine, opt-out HIV screening is consistent with current guidance for PMTCT globally.

Women’s age was also a determinant for screening. The oldest mothers had the lowest chance of being tested. It is possible that health care providers estimate that women of a certain age might not be at risk for HIV. Prevention of different infectious diseases possibly transmitted through and during pregnancy is not subject to the mother’s age [29], and this should not be any different in the case of HIV simply due to stigma or assumptions about HIV risk based on age. 

At the social level, education and economy determined screening rates. There is approximately a 20% difference in screening rates between the least and the most educated mothers. The latter group seems more knowledgeable about the importance of this screening and its relevance both to their and their child’s health. Less educated women, however, might still be living with the fear of HIV-related stigma or just misperceiving their risk and the severity of the disease. This finding goes along with other reports on HIV knowledge and perception in Central America. In El Salvador, only 72% of women of reproductive age know about the test for HIV detection, with more than a 17% urban/rural disparity; less than 50% of women know more than one way to prevent HIV, and less than 66% of rural women know that HIV can be asymptomatic [22]. 

In our sample, employed women tended to be less screened compared to other women. This finding warrants more investigation as we expected that employment would have a positive impact on women’s health-seeking behavior because it empowers them financially and socially. 

Similarly to educational level, screening increased by household monthly expenditure level. This is reflective of the health care barriers encountered by the poor, who should be a primary target for prevention interventions based on the local epidemiology of HIV in El Salvador. More so, this finding goes along with access to medical insurance. If El Salvador aims for an HIV-free generation, screening should be available at no cost for those who choose it. 

At the structural level, the type of health care provider, the health care setting, and the frequency of antenatal care visits were additional determinants in HIV screening disparities. The failure of health care systems to detect HIV infection during pregnancy has been documented in other Latin American countries [31]. Our findings suggest that HIV screening and prevention education should be strengthened for the nurses, physicians, and other caregivers who provide prenatal care so that antenatal prevention opportunities can be optimized [30]. Ideally, all health care providers should be able to perform a screening test, as they are simple and easy to administer and can save many lives. On the other hand, availability and promotion of basic antenatal care services, including HIV screening at the first prenatal visit, should be unified across all types of health care settings in El Salvador [15].

Our findings are subject to recall bias as women were asked to provide information on their last pregnancy, which could have been five years prior to the survey. However, when data were restricted to the last two years, the rate of screening increased by less than two percent. Also, it is possible that participants might be unaware of the tests they received due to their lack of knowledge around laboratory assessments, and providers might have followed an opt-out strategy for screening which was missed by our participants.

A second limitation to our study is that our sample was selected from the areas that had the highest concentration of the poorest populations in El Salvador. However, we made no distinction between participants during the survey recruitment based on economic level.

By screening pregnant women and monitoring and treating those who are infected, MTCT can be reduced and prevented. 

Today, through the provision of appropriate health care to HIV-infected pregnant women during pregnancy and delivery, and for newborns soon after birth, the rate of HIV transmission can be reduced to less than 2%; in the absence of intervention, it can be as high as 25% among the non-breastfeeding population [32,33]. Pregnant women in El Salvador have differential rates of HIV screening. The disparities we found in HIV screening for pregnant women should be alarming to policymakers and health officials who are working hard to fight the HIV epidemic in El Salvador [12]. The goal of an HIV-free generation can be met only if HIV surveillance among pregnant women is strengthened. Ensuring availability, provision, and financial access to HIV screening among all pregnant women equally is the first and the most crucial step toward this goal. This would require eliminating the currently persisting disparities in HIV screening that are hindering the success that has been achieved so far in PTMCT in El Salvador. 

## References

[1] Institute for Health Metrics and Evaluation (2010) GBD Arrow Diagram. Available: http://www.healthmetricsandevaluation.org/gbd/visualizations/gbd-arrow-diagram. Accessed 10 September 2013

[2] De Nieto LE, de Barrientos M, de Lemus AM (1999). Boletin Informativo. El Salvador: Ministerio de Salud Pública y Asistencia Social, Dirección de Planificación en Salud, Unidad de Información en Salud. Available: http://www.salud.gob.sv/archivos/pdf/boletin1999.pdf . Accessed 10 September 2013

[3] De Nieto LE, de Barrientos M, Tamayo A del C, de Lemus AM (2000). Boletin Informativo Sobre Indicadores De Salud. El Salvador: Ministerio de Salud Pública y Asistencia Social, Dirección de Planificación en Salud, Unidad de Información en Salud. Available: http://www.salud.gob.sv/archivos/pdf/boletin2000.pdf . Accessed 10 September 2013

[4] Rosales CA, de Barrientos M, Tamayo A del C, de Lemus AM (2001). Boletin Sobre Indicadores de Salud. El Salvador: Ministerio de Salud Pública y Asistencia Social, Dirección de Planificación en Salud, Unidad de Información en Salud.

[5] Boletin Sobre Indicadores de Salud (2002). El Salvador: Ministerio de Salud Pública y Asistencia Social, Dirección de Planificación en Salud, Unidad de Información en Salud. Available: http://www.salud.gob.sv/archivos/pdf/boletin2002.pdf. Accessed 10 September 2013

[6] Garcia E, de Barrientos M, Tamayo A, del C, de Quevedo R, de Lemus AM (2004) Boletin Sobre Indicadores De Salud. El Salvador: Ministerio de Salud.

[7] Serpas MV, de Barrientos M, Tamayo A del C, de Lemus AM (2003). Boletin Sobre Indicadores De Salud. El Salvador: Ministerio de Salud Pública y Asistencia Social, Dirección de Planificación en Salud, Unidad de Información en Salud. Available: http://www.salud.gob.sv/archivos/pdf/boletin2003.pdf . Accessed 10 September 2013

[8] Boletin Integrado de Indicadores En Salud (2009). Ministerio de Salud. Available: http://www.salud.gob.sv/archivos/pdf/Boletin_de_indicadores_del_Sistema_Nacional_de_Salud_2009.pdf. Accessed 10 September 2013

[9] Nacional de Progreso en la Informe Lucha Contra el SIDA. Seguimiento a la Declaración Política sobre el VIH en 2011 (2012). El Salvador: Ministerio de Salud, Gobierno de El Salvador. Available: http://www.unaids.org/es/dataanalysis/knowyourresponse/countryprogressreports/2012countries/file,68524,es..pdf. Accessed 31 May 2013

[10] Teva I, Bermúdez MP, Ramiro MT, Buela-Casal G (2012). [Current epidemiological situation of HIV/AIDS in Latin America: analysis of differences among countries]. Rev Med Chil 140: 50–58 /S0034-98872012000100007 22552555

[11] World Health Organization (2005) Summary Country Profile for HIV/AIDS Treatment Scale Up. El Salvador World Health Organization Available: http://www.who.int/3by5/support/June2005_cri.pdf. Accessed 10 September 2013

[12] Balcaceres C, Hughes H, de Monroy M, Schneider R (2010) El Salvador HIV/AIDS Assessment and Design. Recommendations for USAID Support to the National HIV/AIDS Response in El Salvador. El Salvador: USAID pp. 2010–2015. Available: http://pdf.usaid.gov/pdf_docs/PNADU522.pdf. Accessed 10 September 2013

[13] Pan American Health Organization (2011) Country Health profile. El Salvador World Health Organization Available: http://www.paho.org/english/sha/prflels.htm. Accessed 12 February 2013

[14] World Health Organization (210AD) PMTCT Strategic Vision 2010–2015. Preventing mother-to-child transmission of HIV to reach the UNGASS and Millennium Development Goals. Moving towards the elimination of pdiatric HIV. Available: http://www.who.int/hiv/pub/mtct/strategic_vision.pdf. Accessed 5 March 2013

[15] Centers for Disease Control and Prevention (n.d.) Strategy 4: Further Decrease Mother-to-Child HIV Transmission. Available: http://www.cdc.gov/hiv/topics/prev_prog/AHP/AHP-Strategy4.htm. Accessed 12 February 2013

[16] Centers for Disease Control (2011) One Test. Two Lives. HIV Screening for Prenatal Care. Available: http://www.cdc.gov/Features/1Test2Lives/. Accessed 5 March 2013

[17] Chin T, Hicks C, Samsa G, McKellar M (2013) Diagnosing HIV Infection in Primary Care Settings: Missed Opportunities. AIDS Patient Care STDS, 27: 392–7. doi:10.1089/apc.2013.0099. PubMed: 23802143. 23802143 PMC3704080

[18] Rodríguez MI, Espinoza Fiallos EA, Menjívar EV (2012) Estrategia para la eliminación de la transmisión vertical de VIH y sífilis congénita S.

[19] Técnico del Equipo Programa Nacional de ITS-VIH-SIDA (2008). El Salvador un paso adelante en la respuesta al VIH/SIDA y Tuberculosis. El Salvador: Programa Nacional de ITS/VIH/SIDA, Ministerio de Salud Pública y Asistencia Social. Available: http://www.portalsida.org/repos/El_Salvador_un_paso_adelante_en_la_respuesta_al_VIH_SIDA_y_TB.pdf . Accessed 10 September 2013

[20] Remis RS, Merid MF, Palmer RWH, Whittingham E, King SM et al. (2012) High uptake of HIV testing in pregnant women in Ontario, Canada. PLOS ONE 7: e48077. doi:10.1371/journal.pone.0048077. PubMed: 23152762. 23152762 PMC3494693

[21] Nieto AI, Sorto S, Avalos V, Jovel M, Alvarado A, et al. (2011). Informe Nacional Sobre El Estado de Situacion Del VIH En El Salvado En Cumplimiento Del Plan Nacional De Monitoreo Y Evaluacion, Ano 2010. El Salvador: Sistema Unico De Monitoreo, Evaluacion Y Vigilancia Epidemiologica Del VIH-SIDA.

[22] Monteith RS, Stupp PW, McCracken SD (2005) Reproductive, Maternal, And Child Health In Central America. Trends and Challenges Facing Women and Children, El Salvador ∙ Guatemala ∙ Honduras ∙ Nicaragua. Atlanta, GA: Centers for Disease Control and Prevention & United States Agency for International Development.

[23] Bannink-Mbazzi F, Lowicki-Zucca M, Ojom L, Kabasomi SV, Esiru G et al. (2012) High PMTCT program uptake and coverage of mothers, their partners and babies in Northern Uganda: Achievements and lessons learned over 10 years of implementation (2002-2011). J Acquir Immune Defic Syndr. doi:10.1097/QAI.0b013e318282d27f. 23274930

[24] Health Resources and Services Administration (2012). HIV Screening For Pregnant Women. U. S. Department of Health and Human Services. Available: http://www.hrsa.gov/quality/toolbox/508pdfs/hivscreeningpregnantwomen.pdf. Accessed 10 September 2013

[25] Branson BM, Handsfield HH, Lampe MA, Janssen RS, Taylor AW, et al. (2006). Revised recommendations for HIV testing of adults, adolescents, and pregnant women in health-care settings. MMWR Recomm Rep 55: 1–17; quiz CE1–4 16988643

[26] Steinberg M, Cook DA, Gilbert M, Krajden M, Haag D, et al. (2011). Towards targeted screening for acute HIV infections in British Columbia. J Int AIDS Soc 14: 39 doi:10.1186/1758-2652-14-39. PMC3169441 21827673

[27] ACOG Committee Opinion No (2008) 418: Prenatal and perinatal human immunodeficiency virus testing: expanded recommendations. Obstet Gynecol 112: 739–742. doi:10.1097/AOG.0b013e318188d29c. PubMed: 18757690. 18757690

[28] U.S. Preventive Services Task Force (2005). Screening for HIV: Recommendation Statement. Agency for Healthcare Research and Quality. Available: http://www.uspreventiveservicestaskforce.org/uspstf05/hiv/hivrs.pdf . Accessed 5 March 2013

[29] Centers for Disease Control and Prevention (n.d.) Preventing Infections in Pregnancy. Available: http://www.cdc.gov/pregnancy/infections.html. Accessed 12 February 2013

[30] Becker J, Tsague L, Sahabo R, Twyman P (2009). Provider Initiated Testing and Counseling (PITC) for HIV in resource-limited clinical settings: important questions unanswered. Pan Afr Med J 3: 4 10.4314/pamj.v3i1.52442 PMC2984289 21532713

[31] Nielsen-Saines K, Watts DH, Veloso VG, Bryson YJ, Joao EC et al. (2012) Three Postpartum Antiretroviral Regimens to Prevent Intrapartum HIV. Infection - New England Journal of Medicine 366: 2368–2379. doi:10.1056/NEJMoa1108275. 22716975 PMC3590113

[32] Cooper ER, Charurat M, Mofenson L, Hanson IC, Pitt J et al. (2002) Combination antiretroviral strategies for the treatment of pregnant HIV-1-infected women and prevention of perinatal HIV-1 transmission. J Acquir Immune Defic Syndr 29: 484–494. doi:10.1097/00126334-200204150-00009. PubMed: 11981365. 11981365

[33] Connor EM, Sperling RS, Gelber R, Kiselev P, Scott G et al. (1994) Reduction of maternal-infant transmission of human immunodeficiency virus type 1 with zidovudine treatment. Pediatric AIDS Clinical Trials Group Protocol 076 Study Group. N Engl J Med 331: 1173–1180. doi:10.1056/NEJM199411033311801. PubMed: 7935654. 7935654

